# Assessment of Family, Peers, and Externalising Behaviour Dimensions in Adolescence: The Proposal of a Comprehensive Instrument (FPEB)

**DOI:** 10.3390/ijerph18052724

**Published:** 2021-03-08

**Authors:** Letizia Caso, Andrea Greco, Eleonora Florio, Nicola Palena

**Affiliations:** 1 Department of Human Sciences, Lumsa University of Rome, Piazza delle Vaschette, 101, 00193 Roma, Italy; 2 Department of Human and Social Sciences, University of Bergamo, Piazzale S. Agostino, 2, 24129 Bergamo, Italy; andrea.greco@unibg.it (A.G.); eleonora.florio@unibg.it (E.F.); nicola.palena@unibg.it (N.P.)

**Keywords:** adolescents, externalising disorders, externalising behaviour, moral disengagement, family relations, peer relations

## Abstract

In the context of externalising behaviour problems, risk factor research (RFR) focuses on risk and protective factors of juvenile delinquency, which can pertain to individual, system, and societal levels. Several instruments aiming at measuring these factors have been developed, but a comprehensive research tool is missing. The aim of the present study was to develop and validate a questionnaire, the “Family, Peers, and Externalising Behaviour in adolescence” (FPEB) as a tool for assessing adolescents’ tendency of externalising behaviour, the quality of relation with their parents, and peer-relations. FPEB was administered to 835 Italian students (36.8% males, age *M* = 13.81, *SD* = 1.54) together with the Moral Disengagement questionnaire to test concurrent validity. Data about socio-demographics and school performance were also collected. An EFA (Promax rotation, subsample A, *n* = 444) resulted in a four-factor structure that was corroborated by a CFA (subsample B, *n* = 388). The factors were “externalising behaviour” (var 13.16%), “peer relations difficulties” (var 11.10%), “Family conflict” (var 8.32%), and “lack of family negotiation” (var 7.11%) and showed good internal consistency (all α ≥ 0.65). There were differences between males and females in the correlational patterns of the four factors. The FPEB factors also showed good concurrent validity: two of the four factors (“lack of family negotiation” and “externalising behaviour”) and the total score of the scale correlated with the “Moral disengagement scale”, whereas peer relation difficulties did not. Further analyses also showed gender differences (except for “peer relations difficulties”) and an association between students’ school performance and “externalising behaviour”, “family conflict”, and the total FPEB scores. We concluded that the FPEB is a tool that is potentially useful to assess risk and protective factors and to plan targeted interventions (focusing on the specific area). Limitations and suggestions for further improvements are also discussed.

## 1. Introduction

Externalising disorders represent a phenomenon that deserves great attention since it is associated with children’s distress, as well as with that of their family and peers [1]. Externalising behaviour problems are a cluster of maladaptive conducts (e.g., antisocial personality disorder), with the core feature of a disinhibited distress that entails a negative impact on the societal environment external to the individual [2]. In fact, externalising conducts can be defined as uncontrolled manifestations such as aggressive, defiant, and impulsive behaviours [3]. Research concerning juvenile delinquency and antisocial behaviour has directed particular attention to risk and protective factors [4,5,6,7,8,9,10], mostly because early intervention is essential and effective in helping reduce punitive measures for children at risk [11]. The importance of prevention is well-established [12,13,14] and elaborating on preventive interventions on the basis of the varied array of risk factors recognized in research literature should be advisable to improve their effectiveness. Understanding the aetiology of offending and violent behaviour is, in fact, believed to be critical in structuring intervention and prevention attempts [15]. It has been suggested that risk factors can pertain to the individual level (biological, cognitive, and emotional aspects), system level (family, peers, school, and neighbourhood influences), and cultural/societal level (poverty, racism, media depiction of violence, diffusion of drugs, alcohol, and firearms, etc.) [16]. Moreover, risk factors can be defined as proximal or distal, depending on whether they are close or distant in time [17].

In view of such complexity that require various interacting levels and areas when approaching the topic of adolescent antisocial behaviour [18], research designs have used many different instruments and measures in order to investigate the relationship between specific risk (or protective) factors and juvenile delinquency (or antisocial behaviour) [4,5,6,15]. Therefore, such complexity is reflected in the variety of instruments and research designs in this specific research area. In fact, it seems that risk factor research (RFR) concerning antisocial and delinquent behaviour in children is characterized by a variety of conceptualizations and measures that hinder a clear understanding of the numerous findings made in the last few decades in this field [17]. For instance, there are numerous studies on bullying and cyberbullying, but gaps in knowledge regarding protective factors have been recently highlighted [19], and research concerning violence in the adolescence often addresses only one context or includes few variables [20].

In order to facilitate research process on this topic, we elaborated on a comprehensive research tool able to merge at least some of the major areas usually taken into consideration in the research of risk and protective factors. More specifically, we focused on the aforementioned “system level”, and we developed an instrument accounting for the most significant areas in which risk (or protective) factors are considered to play their effects in relation to the onset of adolescent deviant behaviour intended as a maladaptive conduct. According to the reference literature, and considering factors that go beyond the individual sphere, such main areas are the family context, the scholastic context, and the relationship with the peer group [4,5,21,22,23,24,25,26,27]. Each of the three areas is multifaceted since it contains specific aspects that are believed to be particularly influential. Concerning family context, there is a scarcity of holistic approaches that gather risk and protective factors in the same study [10]. Following such consideration, the subareas included in the present work are the following: family conflict, family cohesion, authoritarian and punitive parenting style versus an extremely permissive parenting style, involvement in the children’s life, and presence or absence of emotional warmth. Regarding the scholastic context, De Piccoli et al. [25] affirm that the classroom represents a context where the adolescent can experience different kinds of relationships, mostly classifiable into two categories: horizontal and vertical relationships. On the horizontal level, the child is involved in friendships and conflicts with peers (in the context of the scholastic institution), whereas vertical relationships entail respect and responsibility towards teachers and scholastic authority in general. Moreover, according to the authors, vertical relationships in the scholastic context represent a reflection of the relationship between the adolescent and the adult world itself. Lastly, the peer group area addresses relational difficulties, free choice and reciprocity, curiosity and attention to cultural diversity, pro-social tendencies, trust, and to be at ease in communicating. The literature on the topic supports the importance of considering the peer group network among the other areas traditionally connected to adolescent antisocial behaviour. Antisocial friendships represent a risk factor for antisocial behaviour in adolescence [14], and the total level of aggressive behaviour of a single adolescent in the friend network is predictive of the increase in rule-breaking behaviour of that individual over time [28]. On the other side, adolescent antisocial behaviour itself may represent a means of gaining and maintaining status in the peer group [29]. Peer groups are also as a source of possible protective factors. In fact, higher levels of prosocial behaviour among classmates predicted lower levels of future antisocial behaviour in students [30].

Family and peer support are also related to antisocial behaviour [31,32] and self-esteem [33], the latter of which plays a role in antisocial behaviour as well [34,35]. More studies focusing on the effects of positive peer influence on antisocial behaviour are needed [30]. Hence, having an indication of the quality of students’ relationships is useful to detect those adolescents who are at risk of developing and enacting antisocial behaviour.

Various measures focus on the assessment of externalising behaviours, some of which are the Child Behaviour Problems Checklist, the Youth Self Report and the Teacher Rating Form [36], the Strengths and Difficulties Questionnaire [37,38,39], and the Aggression Questionnaire [40]. Numerous studies in the reference literature further explore the linkages between externalised disorders and parenting methods or peer relationships, and some studies combine measures of externalised behaviours with instruments assessing parenting styles [1,3] or peer relationship quality [41,42,43]. Consistently RFR has gained more and more popularity in the attempt to explore the origins of youth delinquency [44]; nonetheless, to the best of our knowledge, an instrument gathering different risk factor categories pertaining to the system level is not available, probably due to the great number of conceptualization proposals existing in the literature [17]. Measures of this inclusive typology have been elaborated on when individual antisocial thinking is taken into account, e.g., the “Development of the Antisocial Beliefs and Attitudes Scale” [45]. Therefore, the novelty of the Family, Peers, and Externalising Behaviour in Adolescence (FPEB) instrument in RFR is represented by its comprehensiveness, and it is particularly useful in research concerning children’s antisocial and delinquent behaviour when proximal risk factors at the system level are taken into account in research hypotheses. The use of a single, multidimensional tool (instead of a battery of questionnaires created separately) is linked to a complex theory in research explaining discomfort behaviours in developmental age, and this could help to identify more targeted and specific interventions. FPEB offers a global assessment of externalising behaviour tendencies and of parent–child and peer relationships quality, which are both related to antisocial behaviour [31,32].

The first explored area is parent–child interactions, whose relevance to the development of children is well-known. Dodge et al. [46] found that lack of parental warmth and support leads to aggressiveness; Baumrind [47] reports that it leads to lack of empathy. Deković et al. [32] suggests that a poor parent–child relationship leads to more externalized problems, and the relationship between antisocial adolescents and their parents may partially be built on lack of intimacy and anger, amongst others. Baldry and Farrington [5] also studied the importance of positive parent–child relationships and found that poor-quality relationships can lead to a “system risk”. This, in turn, can lead to maladaptive and antisocial behaviour. However, parent–child relationships can be studied under several perspectives, some of which are proximal and some distal [32]. For these reasons, the present instrument included items relating to two main theoretical dimensions: family conflict and family negotiation. The second area regarding peer relationship quality is known to play an important role in positive developmental pathways [48,49] and emotional support [50,51]. This becomes clear when considering that adolescents expect comprehension, ease of communication, trust, and honesty from their peers [25]. The externalising behaviour dimension, previously discussed, represents the third and last area explored by FPEB.

All areas are investigated and take into account a proximal stance, i.e., the current state of situations at the time of filling out the questionnaire. This latter aspect makes the questionnaire suitable for use both in cross-sectional and in longitudinal studies.

The present study had three main goals: (i) to explore the factor structure of a novel instrument aiming at measuring externalising behaviour and the relationship between the students, their parents, and their peers using Explorative Factor Analysis; (ii) to confirm the factor structure obtained in the previous step using Confirmatory Factor Analysis; and (iii) to validate the instrument by correlating it with the Moral Disengagement scale, a 32-item tool measuring moral disengagement over eight dimensions, such as “moral justification” and “victim dehumanization” [52,53,54]. The connection between Moral Disengagement and externalising behaviours has been confirmed in recent studies [29,43,55,56]. Moreover, responses on FPEB will be analysed in light of school performance, since the quality of relationships between students, their parents, peers, and teachers is a predictor of school motivation and performance [48,57,58], and in light of differences between male and females. These aspects are important to consider since recent findings highlight the importance of academic self-efficacy and the possible role of gender [59].

## 2. Methods

### 2.1. Participants

In total, 835 students from nine schools in the Bergamo area (Northern Italy) participated in the study. Age ranged from 10 to 20 years old (*M* = 13.81, *SD* = 1.54). About 37% of the students were in the 10–13 age range, and the remaining were older than 13 years old; 36.8% were male students. Grade ranged from sixth grade (*n* = 376) to twelfth grade. Participants reported having no learning or socio-relational difficulties, nor were they referred to social services.

### 2.2. Measures

#### 2.2.1. Development of a New Scale—Family, Peers and Externalising Behaviour in Adolescence (FPEB)

The scale developed by the authors consisted of 45 items and was used to measure students’ relationships (with their parents and peers) and externalising behaviour. All items in the questionnaire were answered on a 5-point Likert-type scale (1 = never, 2 = rarely, 3 = sometimes, 4 = often, 5 = always). The questionnaire assessed four dimensions in three areas. The Family Conflict dimension, including seven items, assessed situations where family members disagreed on a topic or when there was tension in the family (example item: “My parents argue in my presence”). The Family Negotiation subscale, including 13 items, assessed relationship patterns where the parents failed to negotiate with their child about decisions, activities, etc. (example reverse item: “I negotiate with my parents on what I can do in my free time”).

The second area, including 12 items, focused on one main dimension: “peer relation difficulties”, thus considering the quality and type of relationships with one’s peers, as well as their emotional support. An example item is “I am not accepted by my peers”.

The third area, including 13 items, focused on one dimension: “externalising behaviour”, namely maladaptive behaviours directed toward the environment and other people. An example item is “If someone is made fun of, I do it too”.

All the included items were created starting from the available literature on each of the topics, and proposed to participants in form of affirmations with respect to which respondents had to express their grade of agreement [60].

#### 2.2.2. Moral Disengagement

Building on previous studies underlining the good construct validity of the Moral Disengagement questionnaire [52,53,54], students were requested to fill out this 32-item tool [54]. Caprara et al. [52] theorize that the construct of Moral Disengagement includes eight dimensions: moral justifications, euphemistic labelling, advantageous comparison, displacement of responsibility, diffusion of responsibility, distorting consequences, blaming, and dehumanization. All 32 items are measured on a Likert scale ranging from one (I disagree entirely) to five (I agree entirely). This tool was administered to explore the concurrent validity of the new instrument presented in the current study and is described below.

#### 2.2.3. Student Performance Indicators

Student performance was assessed according to (i) end-of-year outcome, i.e., whether the student was admitted to the following grade or not; and (ii) students’ marks. For the latter, we firstly coded a student’s marks for each class (Italian, history, geography, mathematics, science, physics, and foreign language) as “fail” (scores zero to five), “sufficient” (scores six and seven), or “merit/distinction” (scores from eight to ten). Then, we computed a mean score accounting for all classes.

### 2.3. Procedure

The first step was to contact the schools taking part in this study. Target students were presented with the study aims and goals. School authorities and parents authorized children’s participation in the research. Parents also filled an informed consent form for their underage children, whereas students over the age of 18 signed the consent themselves. The whole process was conducted in accordance with the Declaration of Helsinki [61] and with the ethical guidelines for research provided by Italian Psychological Association [62]. Then, Head Teachers worked with the lead researcher to determine suitable classes, dates, and times to administer the questionnaire. 

For the second step, the importance of sincerity when filling the questionnaire was stressed. Anonymity was assured. Students were instructed on how to fill the questionnaire, which was administered by a trained researcher. Participation was voluntary and provided no remuneration. The design was cross-sectional. 

### 2.4. Statsitical Analyses

The analyses were conducted with IBM SPSS Statistics (version 17) (IBM, Chicago, USA), Mplus (version 7) (Muthén & Muthén, Los Angeles, CA, USA) [63], and Jamovi (version 1.2) (The jamovi project, Sydney, Australia) [64].

All 45 items initially included in the scale were explored via IBM SPSS Statistics for their mean, standard deviation, skewness, and kurtosis to check their distribution. In order to ensure normal distribution, see Ref. [65], we excluded all items whose skewness was above 2 and/or kurtosis above 7.

Roth [66] states that when less than 10% of data are missing, regardless of their pattern, Hot Deck can be implemented [66,67,68,69]. Hence, we used this method to deal with missing values. Hot deck replaces missing values with those of a “donor” in the dataset. In short, the method locates cases similar to those with missing values, and it copies values from the former to the latter. In the present analysis “sex” and “age” were selected to locate “donors”. It was not possible to replace missing values for three cases; hence, these were excluded from the analyses. This resulted in a total sample of 832 participants. The total sample was randomly divided into two sub-samples via IBM SPSS Statistics: one to EFA (SAMPLE A, n = 444) and one to CFA (SAMPLE B, n = 388). The sample was not split into two perfect halves because the SPSS function randomly selected approximately, rather than precisely, 50% of the whole dataset.

The Kaiser Meyer Olkin (KMO) test and Bartlett’s test of sphericity were run to assure that the correlational matrix could be used to perform Factor Analysis. KMO (0.84) should be >0.5, and the Bartlett’s test should be significant. EFA with Promax oblique rotation was used to analyse the items on the FPEB (using IBM SPSS Statistics) because there was theoretical reason to believe the extracted factors were related [32,33]. Initially all 45 items were included. Subsequent factor analyses were conducted in a stepwise fashion to eliminate items until a stable factor solution emerged. In order to select the items to be retained, items with loadings < 0.32 and items with loadings > 0.32 on more than one factor were excluded see Ref. [70] because the factor loadings cut-off score of 0.32 (or above) is generally considered substantial. However, determining the number of factors to be retained judging the elbow of a scree plot could reflect a sampling error, whereas the rule “eigenvalue greater than one” tends to retain too many factors [71,72]. For this reason, we also conducted a parallel analysis with Horn’s method [73] using Jamovi Software. 

Confirmatory factor analysis was conducted on SAMPLE B using MPlus software. To adjust the multivariate non-normal distribution, robust maximum likelihood estimation was employed. To determine whether the expected model fit the data, various indices were used [74]. We used the chi-square test statistic, the root-mean-square error of approximation (RMSEA), the standardized root-mean-square residual (SRMR), and the comparative fit index (CFI). RMSEA and SRMR ≤ 0.08, CFI ≥ 0.90, and non-significant chi-square test statistics were interpreted as reasonable fits. 

Cronbach’s Alpha coefficients were calculated to examine internal consistency, and we deemed values > 0.60 as acceptable [75].

To evaluate concurrent validity, we examined Pearson correlations between each scale of the FPEB and Moral Disengagement total scores.

Furthermore, we also used *t*-tests to measure the difference between males and females and the difference between students who were admitted to the following academic year and those who were not. Correlations were run between student performances and scores on the FPEB.

Missing values were treated via listwise deletion in SPSS and full information maximum likelihood imputation in MPlus. 

## 3. Results

### 3.1. Preliminary Analysis

Item response rate was high (MIN: 97.72%; MAX: 99.76%), and the frequency of missing data was low, ranging from 2 (0.24 %) to 19 (2.27%). Average score in response to the items ranged from 1.33 to 4.52 (*SD*_min_ = 0.78; *SD*_max_ = 1.49). When looking at the distribution of the data, three items showed skewness above 2 (“When there is an argument in my family, someone raises a hand”; “My parents physically punish me if I disobey them”; “I hit a classmate because I considered him/her disloyal or uncaring”); hence, they were transformed into their inverse [76].

### 3.2. Factor Structure of the FPEB—Exploratory Factor Analysis

**Figure 1:**
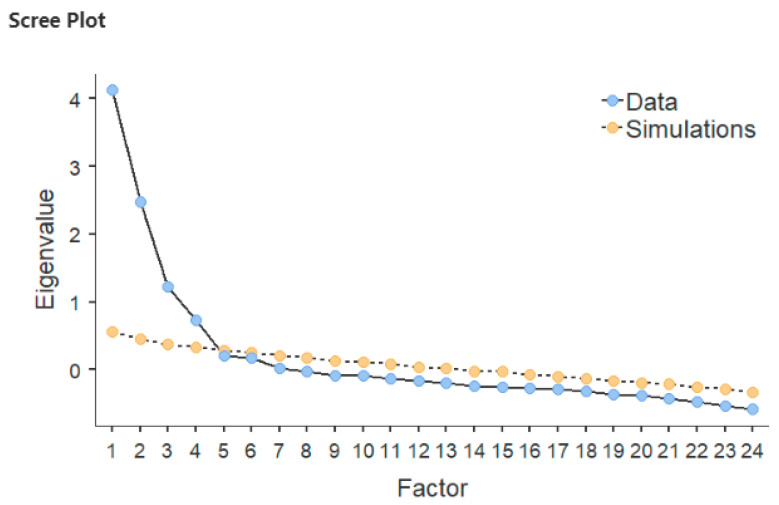
Screeplot of the EFA and parallel analysis.

Data from SAMPLE A, with all 45 items, were used to perform the Exploratory Factor Analysis with IBM SPSS statistics. The Bartlett’s test of sphericity (*χ^2^* = 3062.09, *p* < 0.001) and the KMO (0.84) assured that the correlational matrix could be used for EFA. Examination of eigenvalues and of the scree-plot (Figure 1) suggested a five-factor structure.

After examining internal consistency, one factor showed a Cronbach’s alpha of = 0.55. Hence, the EFA was re-conducted forcing a four-factor structure. The initial pool of 45 general items, after subsequent factor analyses conducted in a stepwise fashion, was reduced to 24 (Table A1). A parallel analysis also indicated that the four-factor solution was the most appropriate (Figure 1). Twenty-one items were excluded because their loadings were lower than 0.32. The pattern of factor loadings is given in Table 1. The first factor explained 13.16% of the variance. It showed loadings from nine items assessing students’ behaviour such as, for example, admission of wrongdoing, lying to parents, and making fun of a victim together with peers. This factor was labelled “externalising behaviour”. The second factor explained 11.10% of the variance after rotation. It showed loadings from seven items assessing students’ relationships with their peers such as, for example, perceiving support from one’s peers, perceiving freedom to express one’s own opinions, and perceived difficulty/easiness to make new friends. This factor was labelled “peer relations difficulties”. The third factor explained 8.32% of the variance after rotation. It showed loadings from four items assessing perceived home atmosphere, such as “my parents argue in my presence” and “I have the feeling that there is a tense atmosphere at home”. This factor was labelled “family conflict”. The fourth factor explained 7.11% of the variance after rotation. It showed loadings from four items assessing the relationships between students and their parents, and in particular to what extent these triads negotiate decision, activities, etc. This factor was labelled “lack of family negotiation”. The model explained in total 39.68% of the variance. As shown in Table 1, no item displayed a loading lower than 0.32. The size of secondary loadings was usually below 0.32.

### 3.3. Factor Structure of FPEB—Confirmatory Factor Analysis

**Figure 2:**
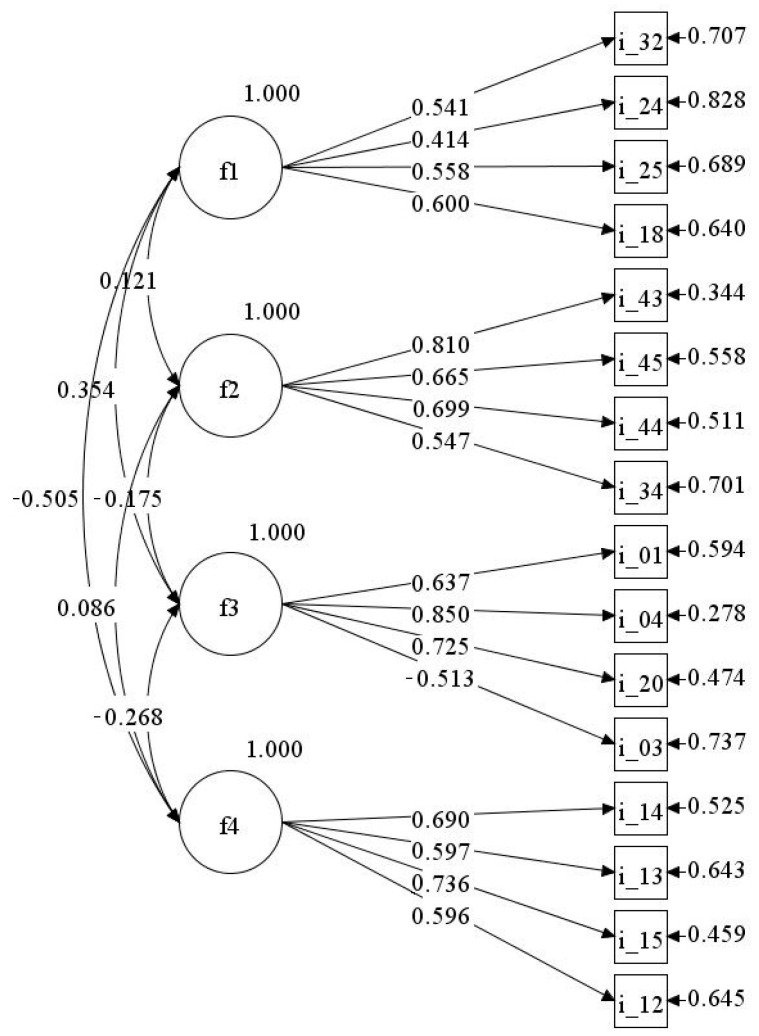
CFA of the Family, Peers, and Externalising Behaviour (FPEB) scale; N = 388. Items i_3, i_12, i_13, i_14, i_15, i_34, i_43, i_44, i_45 are reverse items. f1 = “Externalising behaviour”, f2 = “Peer relations difficulties”, f3 = “Family conflict”, f4 = “Lack of family negotiation”.

Data from SAMPLE B were used to perform CFA. Although we obtained a satisfactory structure with 24 items, the CFA was performed on a 16-item version of the tool, maintaining the four items that showed higher loadings on each of the factors. Indeed, this permits a shorter and more balanced version of the FPEB, which may be particularly useful for students with externalising behaviour. The fit of the CFA model to the data from the 388 students was good (*χ²* (98) = 209.40, *p* < 0.001; RMSEA = 0.054; SRMR = 0.057, CFI = 0.91). Although we outlined a non-significant chi-square as a good model fit, the power of the χ2 statistic is related to sample size, with large samples often yielding significant results. Yet, researchers are advised to always cite the chi-square test [77]. Further, Hu and Bentler [74] suggested that the chi-square statistics should be read in relation to the degrees of freedom, and they state a *χ^2^*/df ratio of less than 3:1 (in our case the ratio was 2.14) suggests good model fit. All items presented significant loadings for their corresponding factors, which were comparable with those found in the EFA, identifying the four factors (Figure 2).

### 3.4. Reliability of FPEB and Correlation among Factors

All factor scores were computed as means of the items composing each specific factor (some items were “reverse items”). All the factor scores showed an acceptable distribution; skewness and kurtosis showed normal distribution (Skewness_MIN_ = 0.33-Skewness_MAX_ = 1.32; Kurtosis_MIN_ = −0.73-Kurtosis_MAX_ = 1.55). The reliability of the factors was good. Internal consistency for “externalising behaviour” was α = 0.67; for “peer relations difficulties” α = 0.78; for “family conflict” α = 0.76; for “family negotiation” α = 0.75. Following guidelines by Cohen [78], we interpreted correlations between factors as measures of effect size. Correlations were considered weak (|0.10| < *r* < |0.29|), moderate (|0.30| < *r* <|0.49|), or strong (|0.50| < *r* < |1|). As shown in Table 2, there was no correlation between externalising behaviour and peer-relation difficulties for female participants, and there was a weak, negative correlation for male participants. In addition, externalising behaviour showed a weak, negative correlation with family conflict and a weak, positive correlation with lack of family negotiation for male participants, whereas such correlations were positive and moderate for female participants. Peer relation difficulties showed a weak, positive correlation with family conflict for both males and females, and there was a weak, positive correlation with lack of family negotiation for female participants only. Family conflict showed a weak, positive correlation with lack of family negotiation for male participants and a moderate, positive correlation for female participants.

### 3.5. Concurrent Validity: Correlation among the FPEB and the Moral Disengagement Scales

The four extracted factors were correlated with the total score of the Moral Disengagement scale (Table 2). Correlations were again considered as weak (|0.10| < *r* < |0.29|), moderate (|0.30| < *r* <|0.49|), or strong (|0.50| < *r* < |1|). The analyses showed that the Moral Disengagement Scale showed a positive and strong correlation with externalising behaviour and a non-significant correlation with peer relation difficulties for both males and females. In addition, the correlation between the Moral Disengagement Scale and lack of family negotiation was significant for both males (weak correlation) and females (moderate correlation), whereas the correlation with family conflict was significant for females only (weak correlation) The computed total score of FPEB showed a positive, moderate correlation with the Moral Disengagement Scale of *r* = 0.38 (*p* < 0.001) for males and of *r* = 0.46 (*p* < 0.001) females.

### 3.6. Relationship between FPEB Scores and Demographics

Several *t*-tests were conducted to explore whether males and females were different on each single factor of FPEB and on its total score. The differences were significant for all factors (Table 2). The results showed that males obtained higher scores than females for all measures, except for family conflict, where the trend was inverted. Furthermore, the difference in total score was also significant, with males showing a higher total score (*M* = 2.15, *SD* = 0.48) than females (*M* = 2.07, *SD* = 0.58); Welch’s *t*(735.88) = 2.15; *p* < 0.05.

We used *t*-tests to explore differences, in total scores and in the four subscales scores, between students who were admitted to the following academic year and those who were not. As Table 3 shows, all comparisons (except for peer relations difficulties) were statistically significant. Further, students who were not admitted to the following academic year showed higher scores than those who were admitted on all variables.

Lastly, we explored the correlation between the total FPEB and its subscale scores and students’ marks. As Table 4 shows, the total score and all subscales, except peer relations difficulties, significantly correlated with students’ marks. All correlations were negative and weak.

## 4. Discussion

The initial aims of the study were to develop a new measure, to test its properties, and to address the hypotheses at the basis of its construction. In the EFA phase, the four-factor model was composed of 24 items and explained 39.68% of the variance. Subsequent CFA was performed on 16 items, those with higher loadings on each factor, in order to simplify the tool and to balance the number of items between dimensions. CFA confirmed the structure emerged in EFA, with all four factors showing acceptable distribution values, good reliability and internal consistency higher than 0.60 [75]. Accordingly, FPEB validation analyses showed the instrument was robust, corroborated by CFA, and was composed of 16 items in its final version. Results mainly confirmed the structure firstly hypothesized on the basis of the reference literature, since the four areas that were initially elaborated were maintained: family conflict and (lack of) family negotiation [5,10,32,46,47], peer relation difficulties [25,48,49,50,51], and externalising behaviour considered in connection with parent–child and peer relationships [14,31,32,79]. The directions of correlations between factors were overall consistent with reference literature: “Externalising behaviour” was positively related to family conflict and to lack of family negotiation. According to our data, externalising behaviour was not significantly related with the subscale regarding difficulties with peer groups for female participants, but it showed a weak, negative relation for male participants, thus indicating the more difficulties in peer relationships are reported, the lower the “Externalising behaviour” score (and vice versa). This result may suggest a particular link between these two variables if we also consider that peer relation difficulties showed a positive correlation with scores on the “Family conflict” dimension; the role of peer relationships for vulnerable youth can assume a paradoxical form, since reduced relations with antisocial peers may indeed represent a protective factor as part of a coping strategy [43], and antisocial behaviours may incur acceptance by deviant peers and rejection by healthy peers [80]. The association with the “Family conflict” dimension could suggest a new set of analyses in future studies that consider Peer relationship quality as a moderator between negative parenting and externalising conducts [59].

“Family conflict” was also positively associated with the “Lack of family negotiation” subscale, which in turn revealed higher scores for those students who were not admitted to the following academic year; this is in line with all other dimensions and with the total FPEB score. This result is consistent with recent literature [80,81]. Reliability of factors was acceptable since *α* was higher than 0.60 [75] in each case.

Correlation between FPEB and the Moral Disengagement measure resulted in the expected direction since a significant, positive correlation was found, both in males (*r* = 0.38, *p* < 0.001) and in females (*r* = 0.46, *p* < 0.001). For this reason, we also highlight the correlation between “Externalising behaviour” and Moral Disengagement (*r* = 0.59, *p* < 0.01). Consistently, it has recently been found that moral disengagement predicts violent behaviours and peer violence [20]. Our results, therefore, encourage to consider FPEB as a tool with sufficient concurrent validity.

School performance, measured in terms of a successful academic year, was significantly associated with “Externalising behaviour” and “Family conflict” dimensions. This is consistent with other recent findings indicating an association between academic performance and antisocial conduct [82], as well as between parent–adolescent conflict and school-related problems such as school disengagement, low academic performance, and negative behaviour in class [83].

Significant differences were found between male and female groups. Males scored higher on the “Externalising behaviour” subscale, in line with the intuitive hypothesis investigated by Burt et al. [84], according to which boys engage more often in antisocial conducts; however, we disconfirm this assumption and suggest the need to be very cautious when interpreting this specific result that should be examined in depth in future studies. Nonetheless, a recent study showed that male gender was a predictor of violence [20]. Moreover, parental disapproval of antisocial behaviour represents an important protective factor against low academic performance for females rather than for males [85]. According to our results, females tended to report both more “Family conflict” and more “Family negotiation” in their domestic environment. This result may indicate females pay greater attention to family climate, which is consistent with other research findings assessing how family processes, including family conflict, were more strongly linked to female adolescent health behaviour [86].

Notwithstanding the encouraging results, this validation study presents some limitations that need to be accounted for: firstly, information about academic performance was self-reported by students. In future studies it might be better to gather such information from more objective sources. Secondly, the instrument was developed in Italian language; therefore, validation in other languages would be needed to compare cross-cultural data. Future studies would also benefit from a longitudinal design, a possibility that unfortunately was not possible in this first phase of instrument validation. Thirdly, it would be interesting to analyse demographics and information about socio-economic status (SES) in depth, as well as individual differences and family conflict/negotiation from the point of view of parents in relation to the dimensions measured by the FPEB Questionnaire. Lastly, the present work represents a preliminary validation step, and it is necessary to perform other validation analyses on the responses from different samples in further research. In this regard, it would be necessary to include clinical and/or forensic samples of adolescents because stronger associations between parenting and externalising conducts have been found in such samples [3]. This could reveal different results concerning our “Peer relations difficulties” dimension, which may be further examined if a measure concerning the respondents’ proximity to antisocial peers is associated with FPEB.

The novel tool developed in this study may respond to the necessity of using validated instruments when considering specific parenting styles in addressing externalising behaviours [1]. This aspect is also linked to the dimension of peer relationships, which have been found to attenuate the link between negative parenting and externalising behaviours in school [59]. This tool can be used in educational settings both to explore the risk of antisocial behaviour externalization in a certain population of children and to generally identify which risk factors are more problematic (e.g., family relationships), thus permitting to design targeted interventions. The FPEB can be used to identify students at risk of developing deviant behaviours. It is possible to plan effective interventions at the individual [87] and public sector levels [88], bearing in mind that early interventions are more effective than late interventions [89]. Additionally, from the perspective of intervention efficacy, adolescents presenting deviant behaviour can be evaluated on FPEB’s dimensions and retested after having benefited from treatment.

## 5. Conclusions

Despite its limitations, this validation study confirms the usability of FPEB to explore the main risk and protective system level factors pertaining to the onset of antisocial behaviour in adolescents. In its actual form, FPEB is suitable to gather data useful in designing research intervention projects in schools, communities, and local social contexts, and this can improve the understanding of specific environmental needs when implementing targeted interventions. According to an evolutionary developmental perspective [90], prevention efforts can be enhanced by further understanding the reasons why a factor protecting against the emergence of antisocial conduct has a positive influence on a specific individual—or on a specific group—in a specific environment.

## Figures and Tables

**Table 1:** FPEB: Final results of Principal Axis Factor Analysis (Promax Rotation); N = 444.

Item	Factor				Communality
	Externalising Behaviour	Peer-Relations Difficulties	Family Conflict	Lack of Family Negotiation	
i_32	**0.694**	−0.022	0.016	0.066	0.46
i_24	**0.632**	0.027	−0.147	−0.103	0.407
i_25	**0.63**	0.012	0.048	0.021	0.409
i_18	**0.583**	0.029	0.072	−0.063	0.407
i_22	**0.584**	0.044	−0.109	−0.069	0.341
i_26	**0.579**	0.061	0.138	0.02	0.395
i_28	**0.548**	0.075	0.037	−0.024	0.327
i_23	**−0.453**	0.061	0.23	0.053	0.197
i_33	**0.464**	−0.118	−0.019	0.144	0.197
i_43 *	−0.036	**0.762**	0.076	0.147	0.59
i_45 *	−0.025	**0.731**	0.081	0.081	0.52
i_44 *	0.069	**0.689**	0.136	0.073	0.446
i_34 *	0.102	**0.571**	0.02	0.018	0.327
i_31	0.129	**−0.509**	0.202	0.183	0.378
i_29	0.098	**−0.485**	0.11	0.06	0.276
i_35	0.152	**−0.425**	0.233	0.188	0.319
i_1	−0.044	0.019	**0.755**	0.04	0.524
i_4	−0.056	0.106	**0.685**	−0.137	0.488
i_20	−0.007	−0.181	**0.574**	−0.114	0.481
i_3 *	0.059	0.004	**−0.542**	0.065	0.301
i_14 *	0.042	0.054	0.001	**0.711**	0.493
i_13 *	0.052	0.014	−0.051	**0.65**	0.424
i_15 *	−0.161	−0.07	−0.089	**0.55**	0.44
i_12 *	−0.081	0.039	−0.16	**0.492**	0.376

Note. ‘Principal axis factoring’ extraction method was used in combination with a promax rotation with Kaiser normalisation. Rotation converged in five reiterations. Cross-loadings items and items loading < 0.32 have been discarded. * reverse item. Bold items indicate factor membership.

**Table 2:** Gender differences and correlations among the Family, Peers, and Externalising Behaviour (FPEB) factors and the Moral Disengagement Scale.

Factors	Externalising Behaviour	Peer Relations Difficulties	Family Conflict	Lack of Family Negotiation	Moral Disengagement Scale	Males	Females	*t*	df
						*M(SD)*	*M(SD)*		
Externalising behaviour ^a^	-	−0.17 **	−0.13 *	0.27 **	0.58 **	2.10(0.83)	1.90(0.74)	3.54 ***	588.05
Peer relations difficulties	0.03	-	0.17 **	0.01	−0.10	1.99(0.76)	1.84(0.77)	2.85 **	816
Family conflict ^a^	0.33 **	0.20 **	-	0.21 **	0.10	1.79(0.73)	1.99(0.89)	−3.57 ***	741.29
Lack of family negotiation	0.39 **	0.12 **	0.38 **	-	0.25 **	2.70(0.99)	2.54(1.06)	2.20 *	816
Moral Disengagement Scale	0.59 **	0.03	0.22 **	0.38 **	-	2.23(.50)	2.05(.44)	5.17 ***	816

Note. * *p* < 0.05, ** *p* < 0.01, *** *p* < 0.001. Above diagonal correlations for males and below diagonal correlation for females. ^a^ Welch’s *t* is used because of the lack of homoscedasticity.

**Table 3:** *T*-tests exploring the differences between students who were admitted to the following academic year and those who were not on the Family, Peers, and Externalising Behaviour (FPEB) factors and total score.

Factors	Admitted	Not Admitted	*t*	df	*p*
	*M(SD)*	*M(SD)*			
Externalising behaviour	2.07(0.82)	2.44(0.91)	−2.42	128	<0.05
Peer relations difficulties	1.72(0.62)	1.90(0.73)	−1.48	128	0.14
Family conflict	2.01(0.93)	2.46(1.09)	−2.54	128	<0.05
Lack of family negotiation	2.53(1.10)	3.18(1.03)	−3.44	128	<0.01
Family, Peers, and Externalising Behaviour (FPEB)	2.08(0.61)	2.50(0.50)	−4.16	128	<0.001

**Table 4:** Correlations among students’ performance and the Family, Peers, and Externalising Behaviour (FPEB) factors and total score.

School Outcome	Antisocial Behaviour	Peer Relations Difficulties	Family Conflict	Lack of Family Negotiation	FPEB
Student’s performance	−0.27 **	−0.04	−0.11 **	−0.24 **	−0.27 **

Note. ** *p* < 0.01.

## Data Availability

The data presented in this study are available on request from the corresponding author. The data are not publicly available due to the participation of underage students.

## Abbreviations

Below we report a list of abbreviations used in the article.

RFR	Risk Factor Research
FPEB	Family, Peers, and Externalising Behaviour (the new tool focus of the present study)
EFA	Exploratory Factor Analysis
CFA	Confirmatory Factor Analysis
RMSEA	Root-mean-square error of approximation
SRMR	Standardized root-mean-square residual
CFI	Comparative Fit Index
SES	Socio-economic status

## Appendix A

**Table A1:** The Family, Peers, and Externalising Behaviour in Adolescence Tool.

i_1	My parents argue in my presence *I miei genitori litigano in mia presenza*
i_3 *	My parents agree with each other when there are decisions to be made *I miei genitori vanno d’accordo quando sono di fronte a delle scelte da prendere*
i_4	In my family any occasion is an excuse to argue *Nella mia famiglia ogni minima occasione è pretesto per litigare*
i_12 *	I agree with my parents on what I can do in my free time *Concordo con i miei genitori ciò che posso fare nel tempo libero*
i_13 *	I agree with my parents on the time I must be back home *Concordo con i miei genitori l’orario di rientro*
i_14 *	I agree with my parents on the number of evenings I can go out *Concordo con i miei genitori il numero di sere che posso uscire*
i_15 *	I agree with my parents on the places I can go *Concordo con i miei genitori i luoghi che posso frequentare*
i_18	I lie to my parents to go out in the evening *Mento ai miei genitori pur di uscire la sera*
i_20	I feel that there is a tense and unquiet atmosphere at home *Ho la sensazione che in casa ci sia un clima teso e poco sereno*
i_22	If a classmate of mine is made fun of, I join in *Se un compagno viene preso in giro da tutti lo derido anch’io*
i_23	I have hit a classmate because I considered him/her disloyal or uncaring *Mi è capitato di picchiare un compagno di classe perché lo consideravo sleale o menefreghista*
i_24	I make jokes about my classmates because it is funny *Mi è capitato di fare scherzi a danno di un compagno perché mi sembrava divertente*
i_25	I do not study a topic if I am uncomfortable with the professor *Mi capita di non studiare una materia se non mi trovo bene con il professore*
i_26	I make fun of professors I do not like *Derido gli insegnanti che non mi piacciono*
i_28	I copy during class assignments *Mi capita di copiare durante i compiti in classe*
i_29	I find it difficult to make new friends *Trovo difficoltà a instaurare nuove amicizie*
i_31	I am not accepted by my peers *Mi succede di non essere accettato dai miei coetanei*
i_32	I do something I know is wrong if my friends do it also *Mi capita di fare qualcosa che so essere sbagliata se lo fanno anche i miei amici*
i_33	My friends encourage me to do things that I do not want to do *I miei amici mi incoraggiano a fare cose che non voglio*
i_34 *	I feel free to express my opinion within my group *Mi sento libero di esprimere la mia opinione all’interno del gruppo che frequento*
i_35	I feel excluded by my friends because I think differently from them *Mi è capitato di essere escluso dai miei amici perché la pensavo diversamente da loro*
i_43 *	I feel accepted by my friends *Mi sento accettato e capito dai miei amici*
i_44 *	I feel I can say anything to my friends *Sento di poter dire qualsiasi cosa ai miei amici*
i_45 *	I can rely on the help of my friends *Posso contare sull’aiuto dei miei amici*

Note. * reverse item.
